# The eSS rat, a nonobese model of disordered glucose and lipid metabolism and fatty liver

**DOI:** 10.1186/1758-5996-2-15

**Published:** 2010-03-17

**Authors:** Stella M Daniele, Silvana M Montenegro, María C Tarres, Juan C Picena, Stella M Martinez

**Affiliations:** 1 Facultad de Ciencias Bioquímicas, Universidad Nacional deRosario, Rosario, Argentina; 2 Facultad de Ciencias Médicas, Universidad Nacional de Rosario, Rosario, Argentina; 3 Consejo de Investigaciones, Universidad nacional de Rosario, Rosario, Argentina

## Abstract

**Background:**

eSS is a rat model of type 2 diabetes characterized by fasting hyperglycemia, glucose intolerance, hyperinsulinemia and early hypertriglyceridemia. Diabetic symptoms worsen during the second year of life as insulin release decreases. In 12-month-old males a diffuse hepatic steatosis was detected. We report the disturbances of lipid metabolism of the model with regard to the diabetic syndrome.

**Methods:**

The study was conducted in eight 12-month-old eSS male rats and seven age/weight matched eumetabolic Wistar rats fed with a complete commercial diet *al libitum*. Fasting plasmatic glucose, insulin, triglycerides, total cholesterol, low-density and high-density lipoprotein, and nonesterified fatty acids levels were measured. Very low density and intermediate-density lipoproteins were analyzed and hepatic lipase activity was determined.

**Results:**

eSS rats developed hyperglycemia and hyperinsulinemia, indicating insulin resistance. Compared with controls, diabetic rats exhibited high plasmatic levels of NEFA, triglycerides (TG), total cholesterol (Chol) and LDL-Chol while high-density lipoprotein (HDL) cholesterol values were reduced. eSS rats also displayed TG-rich VLDL and IDL particles without changes in hepatic lipase activity.

**Conclusion:**

The nonobese eSS rats develop a syndrome characterized by glucose and lipid disorders and hepatic steatosis that may provide new opportunities for studying the pathogenesis of human type 2 diabetes.

## Background

Diabetes mellitus extensively alters lipid metabolism and, in turn, dyslipidemia appears to play an integral role in the development of impaired insulin secretion [1]. Since increased plasmatic triglycerides and decreased high-density lipoprotein are risk factors of coronary heart disease, these changes are relevant to prognosis with serious consequences in terms of morbidity and mortality [2,3], particularly in those patients with long-lasting diabetes [4].

The eSS rat is a spontaneously diabetic model obtained in Rosario, Argentina, by genetic manipulation; the generation has been described in detail by Martinez *et al *[5]. eSS rats develop a mild type 2-diabetes not related to obesity with higher expression in males [5,6]. Until 12 months of age, progressive rising values of fasting hyperglycemia and glucose intolerance are accompanied by higher levels of circulating insulin indicating resistance to the hormone [5,6]. In 5-month-old males, increased plasma triglycerides levels were verified before the onset of fasting hyperglycemia [7,8]. During the second year of life, as hyperglycemia severely worsens, insulin release decreases, islets display a marked disruption of the architecture and the percentage of β cells diminishes [5,6]. In 12-month-old eSS males we reported hepatic steatosis with a pattern of lipid deposits similar to the one observed in biopsies of type 2 diabetic patients [9].

Taking advantage of the naturally slow progress of the metabolic derangement in this model and the consequent long-life-span of the eSS rats, we have performed the study of lipid metabolic alterations in 12-month-old rats analyzing their role in this spontaneous type 2 diabetic syndrome and its complications.

## Methods

### Subjects

Eight 12-month-old eSS male rats and seven eumetabolic Wistar (W) rats, paired by age and sex, were studied. eSS is an inbred rat maintained in the School of Medicine, Rosario University, Rosario, Argentina. W came from the animal breeding facilities in the School of Biochemical Science, Rosario University. Breeding conditions were the same for all the animals, including temperature regulation (24°C) and light-darkness cycles as well as artificial air exchange. In all the cases, the individuals had remained housed since they were 21 days old, in hanging collective cages. All animals were fed on a complete commercial diet, special for laboratory rats, and water was *ad libitum*. These experimental conditions were maintained until the animals were euthanized. All experimental procedures presented in this study were approved by the Bioethics Commission of School of Medicine, which assures adherence to the standards by the Guide for the Care and Use of Laboratory Animals.

### Experimental procedures

Hepatic lipase (HL) activity was measured in post-heparin plasma obtained from femoral artery under sodium thiopental anesthesia (0.35 ml/100 g body weight of 0.5% solution). Heparin (6 IU/100 g body weight) was injected through the femoral vein and after 10 minutes, blood samples were taken, immediately centrifuged for 10 minutes at

-4°C (2,500 rpm) and stored at -20°C until the determination of enzymatic activity. The animals were stitched and housed in individual cages for 48 hours for their recovery.

After 10-hs overnight fast, all animals were exsanguinated by cardiac puncture under deep sodium thiopental anesthesia. Disodium EDTA (1.5 mg/ml) and sodium azide (0.1 mg/ml) were added to inhibit lipid peroxidation and degradation of lipoproteins and bacterial growth. Samples were centrifuged within two hours of blood collection and the plasma kept at 4°C. Within 48 hours, it was split into two aliquots: one was used for the determination of fasting plasmatic glucose (G0), insulin (I0), triglycerides (TG), total cholesterol (Chol), low-density lipoprotein (LDL)-Chol, high-density lipoprotein (HDL)-Chol, and free fatty acids (NEFA); the other sample was employed for separation and analysis of very-low-density lipoproteins (VLDL) and intermediate-density lipoproteins (IDL).

VLDL and IDL were isolated from plasma by sequential ultracentrifugation at 15°C and 105000 × g for 20 h, at densities of 1.006 and 1.025 g/ml respectively. After centrifugation, the lipoproteins were carefully recovered in the supernatant by tubeslicing. The chemical composition of the fractions was evaluated by measuring Chol, TG, phospholipids (PL) and proteins (Pro). VLDL size was estimated by triglyceride/protein (TG/Pro) ratio. Chol versus TG ratio (Chol/TG) was examined in VLDL and IDL in order to estimate the degree of lipolytic process.

G0, TG, and Chol concentrations were measured enzymatically by commercial kits (Wiener Lab, Argentina). PL concentration was determined using the Bartlett assay; total proteins were measured by the Lowry method using a commercial standard kit of bovine serum albumin (Sigma A 6003; Chemical Company). NEFA levels were evaluated by a standardized enzymatic method (Boehringer Mannheim Lab). Intra-trial variation coefficient for Chol was lower than 3%; for TG it was lower than 4%; for HDL-Chol it was 4.3%; for LDL-Chol it was 4.7% and for NEFA it was 3%. Plasmatic Insulin concentration was quantified by radioimmunoassay (RIA) using rat NIH insulin as standard. Hepatic lipase (HL) activity was measured according to Francone *et al *modified method [10]. Intra-trial variation coefficient for HL activity measurement was 8%.

### Statistical analyses

The results are presented as the mean ± 1 standard error of mean (SD). Statistical significance was determined by Student's test. P value less than 0.05 (p < 0.05) was accepted as statistically significant.

## Results

As seen in Table 1, eSS rats displayed higher levels of G0 and could be considered as being severely hyperglycemic, according to DeFronzo *et al *(>140 mg/dl) [11], whereas controls remained euglycemic. However, I0 values in eSS were higher than those of W. The Table also indicates that diabetic rats presented greater levels of NEFA and of TG than those of controls. In eSS, plasma Chol and LDL-Chol concentrations were also higher albeit HDL-Chol levels were lower. Body weight was similar in both eSS rats and Wistar rats.

**Table 1:** Body weight, fasting glycemia, insulinemia, triglyceridemia, free fatty acids, cholesterolemia and lipoproteins in diabetic eSS rats and in euglycemic Wistar rats.

Rats	Bw (g)	G0 (mg/dl)	I0 (μU/ml)	NEFA (mmol/l)	TG (mg/dl)	Chol (mg/dl)	LDL-Chol	HDL-Chol
eSS (n = 8)	421 ± 9.13	165 ± 15.5	38 ± 4.6	1.35 ± 0.07	228 ± 24.5	105 ± 9	44.2 ± 7.8	15.2 ± 0.95

W(n = 7)	424 ± 29.4	99 ± 1.5	20 ± 3.6	0.89 ± 0.02	97 ± 5.5	57 ± 2.16	14.2 ± 7.8	23.1 ± 0.88

p	NS	<0.001	<0.05	<0.001	<0.001	<0.001	<0.001	<0.001

Body weight (Bw), basal plasma glucose (G0), basal plasma insulin (I0), plasmatic triglycerides (TG), free fatty acids (NEFA), total plasma cholesterol (Chol), low-density lipoprotein (LDL)-Chol, high-density lipoprotein (HDL)-Chol, Wistar controls (W).

TG percentage composition of VLDL was higher in eSS rats (eSS: 67.4 ± 6.5% vs. W: 45.9 ± 2.8%; p < 0.001). TG/Pro ratio provides an estimate of the VLDL size and indicated that in diabetic rats VLDL particles were significantly larger (eSS: 7.68+3.63 vs. W: 1.79+0.35; p < 0.001); Chol/TG ratio of VLDL was lower in eSS (eSS: 0.08 ± 0.007 vs. W: 0.11 ± 0.008, p < 0.001).

As seen in Table 2, when considering IDL composition, eSS rats presented a higher proportion of TG and a lower of PL, as compared with controls. While in control rats Chol/TG ratio increased in IDL with respect to VLDL (VLDL: 0.11 ± 0.021 vs. IDL: 0.19 ± 0.006; p < 0.001), in eSS rats this proportion remained almost unchanged (VLDL: 0.08 ± 0.19 vs. IDL: 0.072 ± 0.043; p > 0.05); Chol/TG ratio of IDL was lower in eSS (eSS: 0.072+0.043 vs. W: 0.19+0.006; p < 0.001).

**Table 2:** Intermediate-density-lipoprotein composition in diabetic eSS rats and in euglycemic Wistar rats.

Rats	Chol %	TG %	PL %	Pro %
eSS (n = 8)	4.9 ± 1.0	68.6 ± 1.3	7.5 ± 0.05	19 ± 0.79

W (n = 7)	5.6 ± 0.9	32.6 ± 5.3	31.7 ± 3	30.7 ± 2.7

p	NS	<0.001	<0.001	<0.001

Intermediate-density-lipoprotein (IDL), cholesterol (Chol), triglycerides (TG), phospholipids (PL), proteins (Pro), Wistar controls (W).

Regarding HL activity, differences were not verified between diabetic rats and controls (eSS: 19.5 ± 0.07 μmol fatty acids/ml PPH vs. W: 20.6 ± 1.4 μmol fatty acids/ml PPH; p < 0.05).

## Discussion

Plasmatic triglyceride values obtained in one-year-old eSS rats indicate hypertriglyceridemia (>200 mg/dl) according to the diagnostic criteria from International Lipid Information Bureau [12]. Hypertriglyceridemia is a common finding in patients with altered tolerance to glucose and in type 2 diabetic patients, since the high proportion of TG in VLDL is one of the main components of diabetic dyslipidemia [13,14]. These findings along with increased LDL-Chol and decreased HDL-Chol levels in eSS rats in relation to the controls, suggest that diabetic animals present a mixed or combined dyslipidemia with a IV phenotype, according to Fredrickson's classification adopted by WHO in 1970 [15].

Albeit 12-month-old eSS rats had an excess of fasting insulin, these animals were unable to normalize blood glucose levels indicating insulin resistance [16,17]. In previous studies we described that 5 month-old eSS males had already developed hypertriglyceridemia and hyperinsulinemia although they remained normoglycemic in fasting state [5,7]. These results point out that in eSS rats fasting hyperglycemia would be a later stage in the sequence of events from insulin resistance to overt diabetes, whilst the insulin resistance affects the lipid metabolism beforehand.

Because elevated plasmatic NEFA levels has been implied as a causative factor of insulin resistance by interfering with glucose cellular uptake and by stimulating the hepatic neoglucogenesis [18,19], attention should be exerted in the elevated NEFA concentrations verified in eSS rats. Moreover, chronic high NEFA plasma levels might be a pathogenic factor involved in both progressive decreasing insulin secretion and lowered percentage of β cells detected in eSS rats during the second year of life [5]. Several studies have proposed that chronic exposure to high NEFA induces beta cell dysfunction and beta-cell death by apoptosis [20,21]). The excess of circulating NEFA causes ectopic fat deposition and the activation of multiple inflammatory pathways with intracellular accumulation of toxic lipid-derived metabolites [22]. Interestingly, we have verified in 12 month-old eSS males a pattern of lipidic deposits in hepatocytes similar to the one observed in type 2 diabetic patients as well as a close association between hyperglycemia values and lipid peroxidation [9]. We suggest that in eSS rats the hepatic steatosis should be closely related to insulin resistance, to increased circulating NEFA [23] and to oxidative stress [24]. The hepatic steatosis of eSS rats should increase triglycerides, reduce HDL levels, and increases small, dense LDL [25], and it should be strongly associated with overproduction of large TGL-rich and protein-poor VLDL particles [26,27], a central pathophysiologic feature of the abnormal lipid profile in both human and experimental type 2 diabetes mellitus [28,29]. Insulin resistance of eSS rats could increase the hepatic uptake of fatty acids released by lipolysis of adipose tissue, the intrahepatic synthesis of triglycerides and the overproduction and secretion of VLDL particles that, in turn, leads to the increased plasma levels of TG. New experiments should be undertaken by measuring the hepatic microsomal transfer protein (MTP) mRNA and protein levels together with the measurement of TG synthesis by the liver after Triton X-100 infusion. These experiments are crucial to brighten the genesis of hypertriglyceridemia and hepatic steatosis in this animal model.

In eSS rats, Chol/TG ratio in IDL was similar too in its precursor VLDL, suggesting that the expected lipolytic cascade has not been produced. Hypertriglyceridemia detected in eSS rats would expose IDL and LDL to an excessive interchange of cholesterol esters by TG doing lipoproteins more prone to HL lipolytic activity. HL catalyzes the hydrolysis of TG of IDL and LDL resulting in smaller, denser lipoprotein particles, which are atherogenic in their oxidized form [30,31]. These mechanisms would be responsible for the increased LDL-Chol in eSS rats. More over, taking into account that LDL size correlated negatively with plasma TG and positively with HDL-Chol [32] we argued that LDL particles are small in eSS rats.

Abnormal composition of HDL, with TG enrichment, would also make HDL a better substrate for LH activity [33,34]. In humans, a proatherogenic role for HL has been suggested from the inverse correlation between increased hepatic lipase activity and the plasma levels of the antiatherogenic HDL and the positive correlation with small dense proatherogenic LDL [35]. In eSS rats HL values were similar to those of the controls without increase in relation to insulin resistance. This could be attributed, in part, to the characteristic non-obese diabetes of this model, since it has been observed that in obese patients the larger trunk is a factor associated with higher HL activity [36]. The observation that California mice (*Peromyscus californicus*) develop a type II diabetes mellitus without obesity showing hyperglycemia and a dyslipidemia characterized by TG overloaded VLDL and without modifications of HL values, lends support to our view [37]. Nevertheless, new experiments using antibodies against LH and against lipoprotein lipase are needed to elucidate these aspects in eSS rats.

Although lipid disorders detected in eSS rats are considered as risk factors in the occurrence of vascular disease, atherosclerotic damage has not been detected in eSS rats even in 24-month old animals [38]. The lack of cholesteryl ester transfer protein in rats [39,40], considered to have a major atherogenic role in humans [40], could be implied in the lack of cholesterol deposits in the arteries of eSS rats.

## Conclusion

The nonobese eSS rats develop a glucose and lipid disorder that could aid in studying the interaction between hepatic steatosis, insulin resistance and dyslipidemia. This model could provide valuable opportunities for investigating the pathways to clinical diabetes and its complications.

## Competing interests

The authors declare that they have no competing interests.

## Authors' contributions

SMD participated in the acquisition, analysis and interpretation of data and helped to draft the manuscript. SMM participated in the acquisition, analysis and interpretation of data and helped to draft the manuscript. TMC participated in the acquisition of data and helped to draft the manuscript. JCP participated in the acquisition of data and helped to draft the manuscript. SMM conceived the study, participated in its design, coordination, analysis and interpretation of data and helped to draft the manuscript. All authors read and approved the final manuscript.
